# Oil Recovery Mechanism of Polymer Gel Injection Between Injection Wells and Production Wells to Block the Dominant Channel of Water Flow

**DOI:** 10.3390/gels11050337

**Published:** 2025-04-30

**Authors:** Dong Zhang, Yan Wang, Peng Ye, Shutong Li, Jianguang Wei, Lianbin Zhong, Runnan Zhou

**Affiliations:** 1 State Key Laboratory of Continental Shale Oil, Northeast Petroleum University, Daqing 163318, China; zhangdong084@163.com (D.Z.); weijianguang@163.com (J.W.); zhourunnan1223@163.com (R.Z.); 2 PetroChina Daqing Oilfield Co., Ltd., Daqing 163458, China; yepeng@petrochina.com.cn (P.Y.); zhonglianbin@petrochina.com.cn (L.Z.); 3 Exploration and Development Research Institute of Changqing Oilfield Company, Xi’an 710018, China; lisht_cq@petrochina.com.cn

**Keywords:** gel system, deep fluid diversion, enhanced oil recovery, pressure gradient, sweep volume

## Abstract

Gel system profile control and flooding is a novel profile control technology designed to address the issue of inefficient and ineffective water circulation in high water cut reservoirs during their later stages, demonstrating significant development potential. This system expands on the swept volume and enhances oil displacement efficiency, ultimately improving oil recovery. In this study, a new “injection well + intermediate well” configuration was employed to conduct physical simulation experiments on core modules using the gel system (Partially Hydrolyzed Polyacrylamide + Cr^3+^ cross-linker + Stabilizer). By adjusting the gel system dosage and the location of the intermediate well (0.123 PV + midway between the injection and production wells), changes in the recovery rate, water cut, seepage field, pressure field, oil saturation field, and swept volume were observed. The experimental results indicate that under these conditions, the model achieved the highest total recovery rate, with optimal displacement of remaining oil. Additionally, the gel system exhibited strong stability after formation and was resistant to breakthrough. Compared to single-injection well profile control and flooding, the configuration increased the recovery rate by 16.7%, demonstrating promising development prospects and application potential.

## 1. Introduction

During the entire production process of an oil field, water flooding is the main technical means for extracting oil and gas. However, as production enters the later stage, conventional water flooding has become difficult to enhance the oil and gas recovery rate [1,2,3]. This is mainly due to the complex heterogeneity of different formations, which will cause fingering during water flooding [4,5,6]. The injected water will drive oil along the dominant seepage channels, and it becomes difficult to reach the “dead oil zone” [7,8,9], resulting in high water saturation in the later stage of the oil field, reducing the oil and gas recovery rate, and increasing the development cost in the later stage, along with other negative effects [10,11,12].

Therefore, in the later stage of development, profile control in injection wells and water shutoff in production wells are usually adopted as the main measures to improve the recovery rate [13,14,15]. Although these two measures can increase the recovery degree of a small part of the remaining oil in the oil field, repeated water injection for oil displacement will cause the flow channels in the high-permeability layer sections to be expanded by water scouring, further increasing the heterogeneity of the reservoir. Even if water injection development continues, it is an ineffective cycle and increases the difficulty of extracting the vast majority of the remaining oil [16,17,18]. In response to this, a new profile control method has been proposed, namely, deep fluid flow diversion [19]. The core of the idea of deep fluid flow diversion in the formation lies in developing a new diversion agent rather than a profile control agent. A flexible diversion agent has been developed, changing the development direction of traditional profile control and water shutoff. By adding the diversion agent in the deep formation to block the high-permeability seepage channels and forcing the injected water to transfer to the medium- and low-permeability layer sections to achieve the purpose of fluid flow diversion, the swept volume is further expanded, and ultimately the recovery rate of oil and gas is improved [20,21]. The proposal of this theory has effectively solved the thorny problem of ineffective circulation of the injected fluid flow during the profile control and water shutoff period, opened up a new path for the research process of profile control and water shutoff, and laid the foundation for the subsequent research of scholars. On this basis, Ma et al. and Wu et al. conducted a series of mechanical performance evaluations on the flexible diversion agent and found that it can adjust itself according to its own flexible deformation properties in different pores to facilitate deep migration to plug porous media. The good deformation ability of the flexible diversion agent and the feasibility of deep fluid flow diversion were demonstrated through uniaxial tensile and compressive experiments, and good results were achieved in field tests [22,23]. In addition, Zhu et al. further enhanced the thermodynamic performance of the gel system and developed a gel diversion agent formula that still remained stable under the conditions of abnormally high temperatures and high salinity in oil reservoirs [24]. Meng et al. proposed a deep fluid flow diversion system suitable for this block by analyzing the mechanism of deep fluid flow diversion of the flexible diversion agent and combining the characteristics of the Bohai Oilfield [25,26]. The combined use of the two systems uses a high-strength gel system to block large pores and fractures macroscopically to increase the resistance along the water flooding path. Microscopically, through the cyclic process of temporary plugging—breakthrough—re-temporary plugging—re-breakthrough of the water flow channel and increasing the resistance of the large pores, the subsequent working fluid is forced to divert [27]. The combined system has a good oil washing effect and the function of reducing the interfacial tension. It can be seen that compared to a single system, the combined system has more obvious advantages and more significant effects in oil displacement. The form of the combined system can also be applied in multi-well cooperation to form a situation of multi-directional improvement of oil displacement efficiency. Deeper profile control requires a large amount of plugging agents. Under a long shear distance, it is difficult to achieve a good plugging effect only relying on the deformation properties of the plugging agent itself. For this reason, Liu et al. carried out the idea of gel dam formation in horizontal wells for deep fluid flow diversion. Taking the positive rhythm thick reservoir of the river as an example, the bottom high-permeability zone was scoured by water flow for a long time, expanding the heterogeneity between layers. Even with water injection profile control technology, the water flow bypass phenomenon in the shallow part is still serious. Therefore, it is necessary to run a horizontal well to form a gel dam system in the deep dominant seepage area to force the fluid to flow into the upper medium- and low-permeability layers. By applying physical, numerical simulation, and CT imaging techniques to evaluate the water flooding effect of different parameters, it is proposed that the key parameters affecting the production degree of medium- and low-permeability reservoirs mainly include the position, height, combination form, and injection timing of the gel dam. Selecting appropriate parameters can effectively improve the water flooding effect [28,29,30]. Relevant petroleum workers have also improved the theoretical technology according to the specific conditions of their respective blocks in laboratory experiments and field applications and put it into use, and they received good feedback on the effect [31,32]. In recent years, Xiong et al. analyzed and summarized the current status and countermeasures of deep fluid flow diversion and profile control and flooding technologies, and they pointed out the existing problems of deep fluid flow diversion and profile control and flooding technologies, such as “the lack of distribution and quantitative description technology of dominant water flow channels, the lack of optimization design methods for deep fluid flow diversion and profile control and flooding, the inability of existing technologies to completely achieve true deep placement, the lack of low-cost and long-acting chemical agents, and the lack of monitoring methods for deep pressure and flow fields” [33].

**Figure 1:**
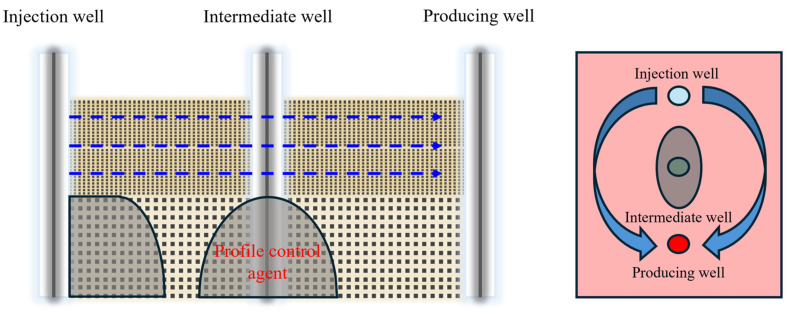
Schematic diagram of the combined profile control of the gel system of “injection well + intermediate well”.

According to previous scholars’ research, it is known that when using profile control technology for injection wells and water shutoff technology for production wells, the plugging of dominant seepage channels is only effective in the near-wellbore area. If the plugging agent is to migrate to the deep part, a larger dosage is required, which not only increases the cost but also aggravates the damage to the formation environment. Moreover, near the injection and production wells, the pressure gradient is relatively large, and the injected water may break through the gel system and return to the dominant seepage channels, making it difficult to achieve the purpose of improving the swept volume. The gel-forming dam technology in horizontal wells is mainly applied in thick oil layers and is a good choice for oil fields with good productivity and large reserves. However, for the vast majority of oil reservoirs, the cost of horizontal wells is relatively high, making it difficult to obtain good economic benefits. As shown in Figure 1, with deepening development, due to poor development benefits, many abandoned wells will appear in the oil field. By utilizing the abandoned wells and combining them with injection wells in the form of an “injection well + intermediate well”, a new profile control combination can be formed. In this form, adding the profile control agent in the intermediate well can effectively prevent the injected water from breaking through to the dominant channels in the deep formation, stabilize the streamline trend, and reduce the interwell fracturing gradient, thereby further improving the recovery degree. This new combination form can not only achieve the purpose of deep fluid flow diversion but also “turn waste into treasure” for abandoned wells.

**Figure 2:**
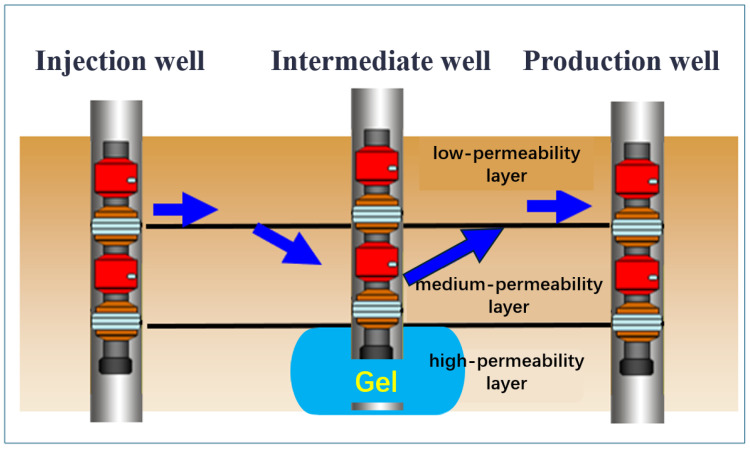
Schematic diagram of deep fluid flow diversion of gel system between injection and production wells.

Gel profile control and flooding technology is one of the important methods for improving the recovery rate of waterflooding reservoirs in oilfields. This method utilizes the abandoned wells between injection wells and production wells. By adding the gel system in the abandoned wells for deep profile control and flooding to form gel wells, it can adjust the fluid flow direction in the deep part of the reservoir, prevent the area of dead oil zones from further expanding, and thereby achieve the goal of improving the recovery rate [34,35,36]. As shown in Figure 2, The pressure gradient in the middle of the injection and production wells is relatively low. The gel system has high stability and is not easily dispersed. At that point, the viscosity of the high-permeability layer section is high, and the flow resistance is stronger than that of the middle and lower sections. It is difficult for the subsequent injected water to continue advancing under the action of this system, so this will cause the middle- and low-permeability reservoirs to form new flow channels, thereby effectively blocking the dominant seepage channels, enabling the utilization of reservoirs with poor physical properties, controlling inefficient and ineffective circulation, reactivating the highly dispersed remaining oil after polymer flooding, and improving the oil displacement efficiency [37,38].

This research aims to analyze the mechanism of deep profile control and flooding in the “injection well + intermediate well” combined form, further analyze and optimize the factors that affect improvement of the recovery rate explored by previous scholars in deep profile control, and adjust key parameters through laboratory experiments to achieve a good oil displacement effect.

## 2. Results and Discussion

This experiment mainly focuses on the influence of single-injection well profile control and drive and the combined profile control and drive of gel systems on the development effect, as well as the influence of the gel system dosage (gel pre-placement radius) and gel well location effect between injection and production wells. A total of six groups of controlled experiments were designed. The specific experimental schemes are shown in Table 1.

### 2.1. Recovery Rate and Water Cut

**Figure 3:**
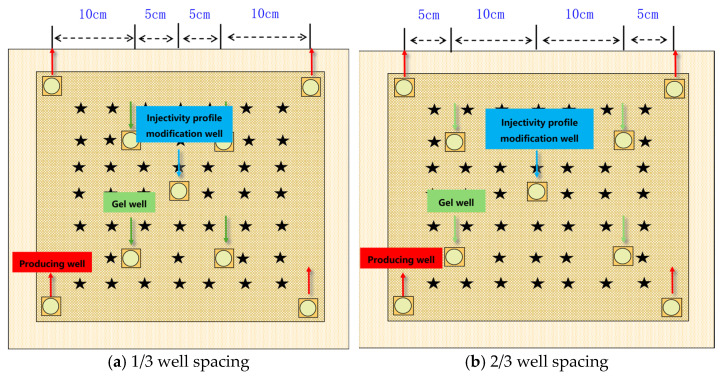
The well layout methods of the intermediate well at different distances from the injection well.

An experiment was conducted using a 30 × 30 × 4.5 cm^3^ flat plate core model to simulate the enhanced oil recovery effect of the “injection well + intermediate well” gel system combination profile control and drive in the area between injection and production wells. The schematic diagram of well placement in the model is shown in Figure 3. During the experiment, the oil production, water production, pressure, and resistance at each test point of the production well were recorded. The recovery rate and water cut were calculated, and the planar distribution cloud diagrams of the pressure field, seepage field, and saturation field of the model were drawn.

**Figure 4:**
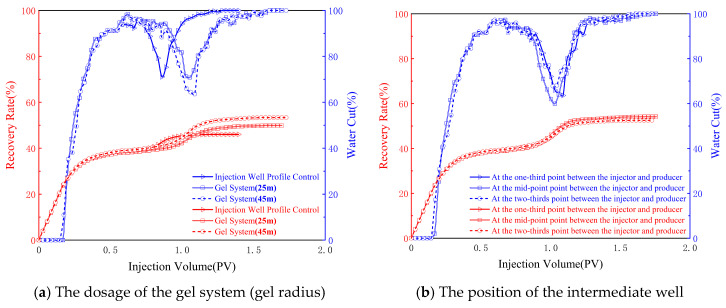
The variations in recovery rate and water cut with injection volume under different parameters.

The model was subjected to water injection development until the water cut of the production well reached 95%. The gel system of the intermediate well followed a specific formula (0.0628/0.123/0.203 PV + gel system of the injection well 0.15 PV + subsequent water flooding until the water cut reached 98%). The variations in the recovery rate and water cut with the injection volume PV are plotted in Figure 4.

The initial water cut showed a sharp upward trend. After gelation, when subsequent water flooding was conducted, the water cut decreased significantly, and in the later stage, the water cut gradually rose back to 100%. Under different parameters, the variations in the recovery rate and water cut presented different effects. When controlling the dosage of the gel system, the total recovery rates were 46.0%, 49.9%, and 53.7%, respectively. Compared to single-injection well profile control and flooding, using the intermediate well combined profile control and flooding system could effectively delay the breakthrough time of the plugging system. The combined system profile control and flooding increased the total crude oil recovery rate by 3.9% and 7.7%, respectively. The water cut decreased to 71.1%, 70.9%, and 60.1%, respectively, during the profile control and flooding process. When changing the position of the intermediate well, the overall decline in the water cut was better, which could effectively improve the degree of crude oil recovery. When the position of the intermediate well was at 1/3, 1/2, and 2/3 between the injection well and the production well, the total crude oil recovery rates were 53.4%, 54.2%, and 52.5%, respectively, and the water cuts decreased to 63.3%, 59.9%, and 67.8%, respectively (Table 2). When changing the position of the intermediate well, both the recovery rate and water cut also changed. Among them, when the intermediate well was in the middle between the injection well and the production well, the recovery rate was the highest, which was 8.2% higher than that of the single-injection well profile control and flooding method. Secondly, the increase in the total recovery rate was greater when it was closer to the injection well than when it was closer to the production well. The overall water cut showed a decreasing trend.

The experimental data reveal a three-stage water cut variation pattern of “sharp rise—sudden drop—gradual recovery” (Figure 4a), demonstrating the dynamic plugging–breakthrough process of the gel system. The initial water cut increase reflects rapid water front advancement, while the post-gelation dramatic reduction (minimum 60.1%) confirms effective high-permeability channel blocking, diverting subsequent fluids to unswept zones such that the gel system dosage must be carefully optimized—excessive injection leads to undesirable coverage of remaining oil along the main flow channels, impairing recoverability, while insufficient dosage fails to establish effective in-depth reservoir plugging. An appropriately designed gel volume can effectively mitigate premature water breakthrough through preferential flow paths while minimizing the obstruction of residual oil saturation. Notably, the time required for the water cut to ultimately recover to 100% shows a positive correlation with the gel dosage, indicating that system strength determines plugging duration—the characteristic recovery pattern results from progressive gel network degradation under prolonged flushing.

The peak recovery rate of 54.2% observed when the intermediate well was positioned at the 1/2 interwell location (Figure 4b) carries significant engineering implications. The simulation results demonstrate that this optimal placement creates symmetric pressure distribution, maximizing the gel’s radial conformance. In contrast, 1/3 or 2/3 placements generate asymmetric pressure gradients that preferentially plug near-well regions, leaving distal oil inadequately mobilized. This deviates from traditional linear displacement theory’s monotonic prediction, proving the existence of an optimal well placement–recovery relationship.

### 2.2. Seepage Field and Pressure Field Distribution

**Figure 5:**
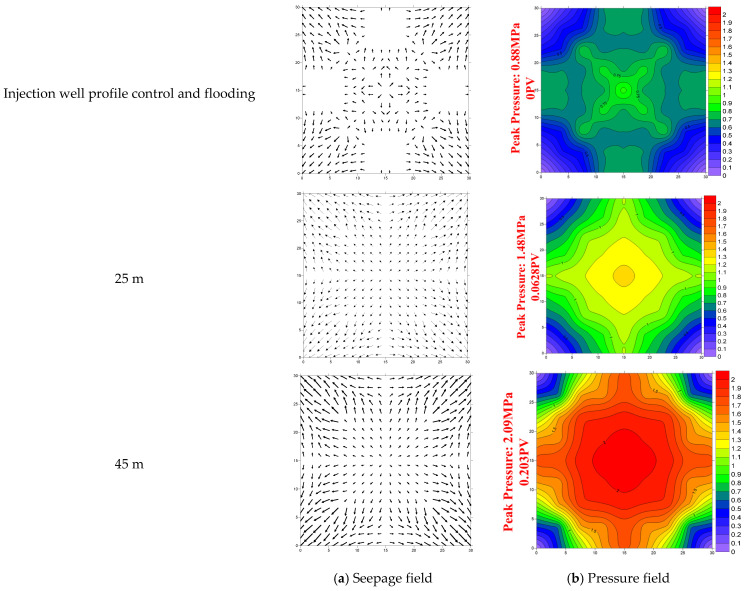

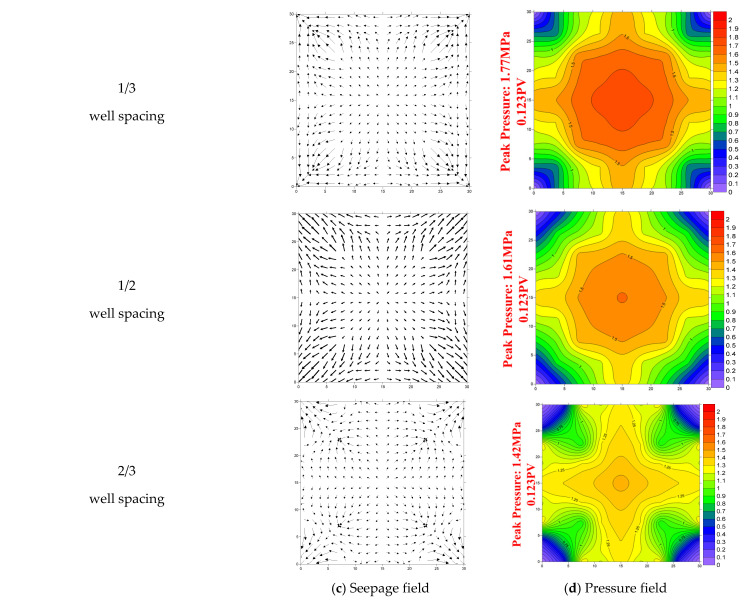
(**a**,**b**): The pressure field distribution under different dosages of the gel system. (**c**,**d**): The pressure field distribution under the condition of different positions between the injection and production wells.

The pressure field of the model after employing the combined profile control and flooding of “injection well + intermediate well” is plotted as shown in Figure 5.

During the water flooding process, the injection pressures corresponding to three different gel system dosages in the models were only 0.06 MPa, 0.075 MPa, and 0.070 MPa, respectively. After applying the combined system profile control and flooding, the injection pressures increased significantly to peak values of 0.88 MPa, 1.48 MPa, and 2.09 MPa. When the location of the intermediate well was altered, the pressures during water flooding in the three corresponding models were 0.065 MPa, 0.065 MPa, and 0.072 MPa, respectively. After applying the combined system profile control and flooding, the injection pressures rose sharply to peak values of 1.77 MPa, 1.61 MPa, and 1.42 MPa.

In the early stages of water flooding, the pressure distribution was uneven, with gradual frontal advancement along the path from the injection well to the production well, leaving a large unswept area. After applying the “injection well + intermediate well” combined system, the interval between the injection well and the gel-treated well exhibited improved displacement efficiency, with streamline fields diffusing toward the flanks of the model.

Excessive gel system dosage resulted in abnormally high pressure near the injection well, while insufficient dosage failed to effectively reach the dead oil zones far from the dominant flow channels. An appropriate gel system dosage could effectively mitigate the uneven planar pressure distribution, maximizing the sweep efficiency between the injection and production wells.

When the gel-treated well was positioned too close to the injection well, fluids bypassed the gel-treated well and tended to converge back toward the main flow channels, leaving a significant unswept volume due to streamline convergence, thereby impairing development efficiency. Conversely, when the gel-treated well was placed too far from the injection well, the profile control near the injection well only improved the streamline distribution in the near-wellbore region, with streamlines quickly reverting to the main flow path, limiting the expansion of the swept volume in the reservoir.

In summary, an optimal gel system dosage combined with positioning the intermediate well at the midpoint between the injection and production wells resulted in the most uniform streamline distribution and the best displacement efficiency, thereby achieving the highest oil recovery rate.

### 2.3. Oil Saturation Field Distribution

**Figure 6:**
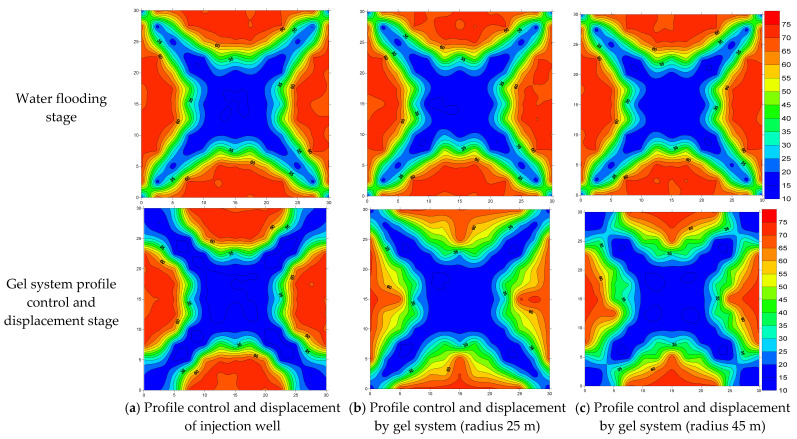

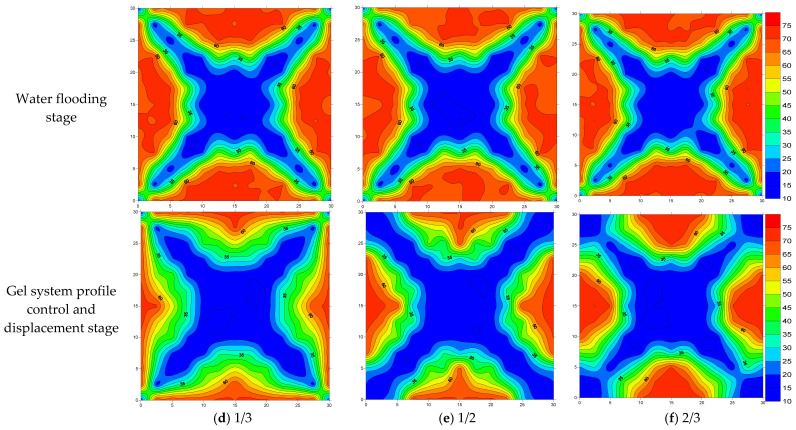
(**a**–**c**): The distribution of the oil saturation field under different dosage conditions of gel systems. (**d**–**f**): The distribution of the oil saturation field under the condition at different positions between the injection and production wells.

In this paper, the microelectrode core model was used to measure the resistance of cores with different permeabilities under different oil saturation conditions, and the standard curve of the rock–electric relationship was established. During the displacement experiment of the electrode model, by systematically monitoring the resistance at different test points, the oil saturation value of each test point could be inversely calculated based on the standard curve. The distribution of oil saturation in the model at a certain moment was plotted by the SUFER software (8.0). The distribution cloud maps of oil saturation under different well spacings are shown in Figure 6.

Analysis of the initial oil saturation distribution cloud maps reveals an average original oil saturation of 70.5% across all six models. Water injection established dominant flow channels extending from injectors to producers, with significantly wider channels observed near injection wellbores compared to production wellbores. This indicates more thorough sweep efficiency in near-injector regions, while deeper reservoir zones require improved conformance control.

The main flow channels exhibited effective oil displacement with relatively low residual oil saturation. However, displacement efficiency progressively deteriorated toward the model flanks along the mainstream lines, leaving unswept dead oil zones in areas with inadequate waterflood coverage. Following conformance control treatment, the gel system demonstrated enhanced local sweep efficiency between injectors and gel placement wells by increasing flow resistance. The primary mechanism involves viscosity elevation after gelation, which substantially reduces water mobility through dominant flow paths and forces subsequent injected water to divert into unswept zones. The unswept dead oil zones pose significant constraints to long-term reservoir development efficiency. These bypassed oil areas not only directly reduce ultimate recovery rates but also induce secondary damage, including crude oil densification, pore throat blockage, and wettability reversal. These combined effects increased the costs of subsequent stimulation measures by 2–3 times. Through residual oil regulation, the activation efficiency of dead oil zones can be substantially enhanced, which is critical for maximizing economically recoverable reserves.

### 2.4. Expand the Swept Volume and Improve the Oil Washing Efficiency

**Figure 7:**
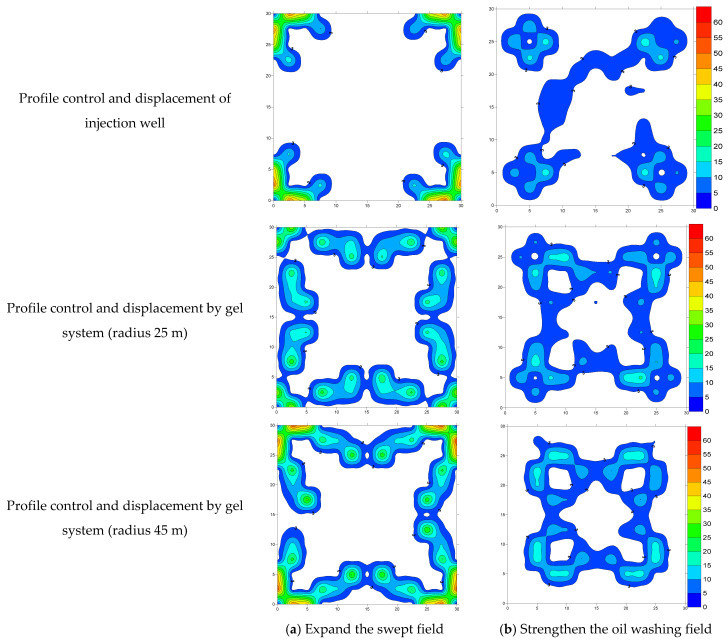

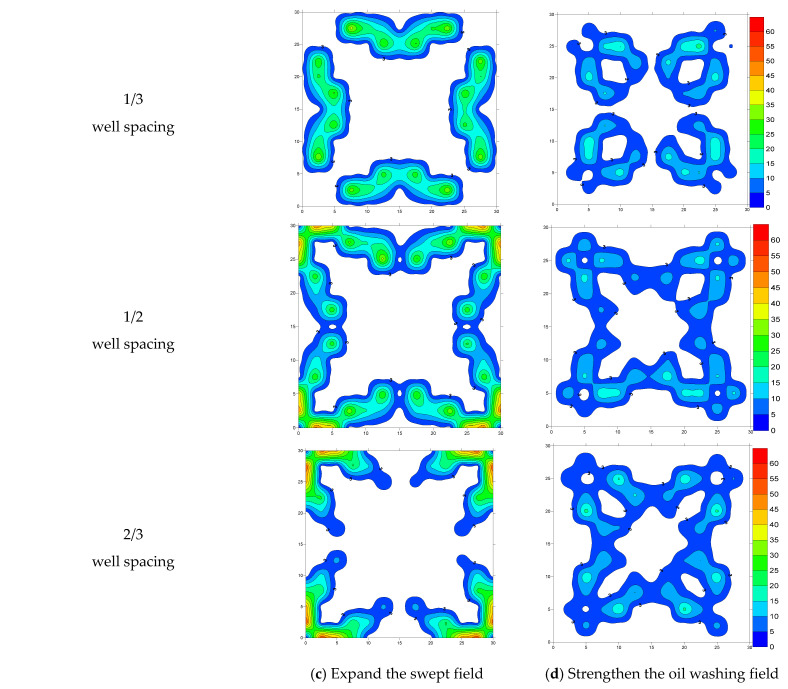
(**a**,**b**): Cloud chart of the difference in oil saturation under different dosage conditions of gel systems. (**c**,**d**): Cloud chart of the difference in oil saturation under the condition of different positions between injection and production wells.

Statistical analysis was conducted on the grids where the oil saturation field changed. To further compare the influence of the gel well location on expanding the swept volume and improving the oil washing efficiency, in this study, the final saturations of the models at different displacement stages were subtracted to reflect the effect of the “injection well + intermediate well” on the remaining oil. Cloud charts of expanding the swept volume and enhancing the oil washing effect under different development schemes were drawn, and the results are shown in Figure 7.

By comprehensively comparing the expanded swept field and the enhanced oil washing field, it can be understood that the profile control and displacement effect of a single injection well is relatively poor, and there is still a large area of remaining oil in the area, with a large unswept area and a low oil washing efficiency. In the case of a small dosage of the gel system, the initial water flooding only evenly displaces the remaining oil in the zone near the injection well. The subsequent water flow will re-circulate from the middle zone back to the main streamline, the ability to produce the remaining oil in the middle and deep zones of the reservoir is poor, and the water flooding effect is only slightly improved. However, although a higher dosage of the gel system can effectively control the bypassing in the central area, the bypassing problem in the deep area of the reservoir is still difficult to be properly solved. Based on our results, the expanded swept areas were 16.7%, 25.0%, and 27.8%, respectively, and the oil washing efficiency improvement rates were 2.3%, 7.9%, and 7.8%, respectively.

When the well placement is at a distance of 1/3 of the well spacing from the injection well, the expanded swept area reaches 25%, and the oil washing efficiency improvement is 33.4%. It can effectively delay the rapid backflow of water to the main streamline in the later stage, and the swept area also has a better improvement. Moreover, if the well placement shifts toward the production well, the influence degree will be greater. When the well placement is at a distance of 2/3 of the well spacing from the injection well, the expanded swept area is also 25%, and the oil washing efficiency improvement is 32.6%. The swept capacity and oil washing efficiency are worse than those at the other two positions, but overall, they show an improvement trend. The statistical results of the expanded swept volume and the improved oil washing effect clearly indicate that the optimal gel system dosage is 0.123PV. When the placement of the gel well is at 1/2 of the distance between the injection and production wells, the effects of expanding the swept volume and improving the oil washing efficiency are the most significant. The water flow spreads evenly in all directions, and both the swept area and the oil washing efficiency are in the best state, which can effectively enhance the crude oil recovery rate. Among them, the expanded swept volume is 27.8%, and the oil washing efficiency improvement reaches 37.0%.

Low-concentration gel systems (<0.123 PV) only induce localized streamline adjustments near the injection well, causing subsequent displacing fluids to bypass back into high-permeability channels (main flow paths). This indicates that low-strength gels are insufficient for achieving deep fluid diversion, which aligns with the “near-wellbore plugging–early breakthrough” phenomenon reported in previous studies.

High-concentration gel systems (>0.123 PV) can effectively mitigate bypassing in the middle zone but show limited improvement in sweep efficiency for deep low-permeability regions, likely due to restricted gel migration or reservoir heterogeneity. These results demonstrate that merely increasing the gel concentration cannot fully resolve deep fluid bypassing issues, necessitating integration with deep fluid diversion techniques (e.g., in-depth gel treatment or nanoparticle-assisted systems).

Placement at 1/3 of the well spacing enhances near-wellbore streamline uniformity but leads to premature breakthrough due to production well offset, resulting in only marginal incremental oil recovery (33.4% vs. 37.0%). In contrast, optimal gel placement at 1/2 of the well spacing achieved the best balance between sweep volume (27.8%) and displacement efficiency (37.0%). This confirms that centrally positioned gels maximize interference with dominant flow paths, forcing fluid diversion into low-permeability zones. Midpoint placement also promotes balanced pressure distribution, thereby delaying water channeling.

Experimental data reveal that sweep volume expansion (27.8%) contributes more significantly to recovery than displacement efficiency improvement (37.0%), highlighting that the primary mechanism of gel-assisted flooding lies in flow field modulation rather than direct interfacial tension (IFT) reduction. This contrasts with conventional chemical flooding (e.g., surfactant-based methods), which primarily relies on IFT alteration for oil mobilization.

### 2.5. Limitations of the Gel System

Generally, the requirements of gel systems in field applications are high adhesive strength, long gel migration distance, and long gel system duration. However, these requirements are difficult to control to produce an optimal solution. In order to move the gel system far, it is necessary to reduce the viscosity of the gel system, though reducing the viscosity will lead to low strength of the gel system after formation, and the strength will affect the maintenance time of the system. Therefore, we chose to use intermediate wells to balance the relationship between the three. Intermediate wells can effectively solve the problem of the gel system moving to the deep part of the reservoir, which can solve the urgent need at present, but it is also limited to the situation where there are redundant abandoned wells. It is hoped that the plugging capacity of the gel system can be regulated in a reasonable way in the subsequent research content.

## 3. Conclusions

In response to issues such as the large number of layer series well patterns, complex mining objects, severe reservoir heterogeneity, and the gradually deteriorating effect of water control and potential tapping measures during the ultra-high water cut period of water flooding, a method of injecting gel system combinations for profile control and plugging of dominant seepage channels in “injection well + intermediate well” is proposed. In this paper, by calculating the distribution characteristics of the pressure gradients between injection and production wells, the distribution characteristics of the pressure gradients between injection and production wells under different conditions are clarified. The gelation and plugging performance and erosion resistance of the gel system were carried out using sand-filled tubes, and the parameter optimization of the combined profile control and flooding of the gel system in “injection wells + intermediate wells” was carried out using a flat core model. The conclusions are as follows:

(1). When the gel injection well between the injection and production wells is at a relative distance of 1/2 from the injection well, the pressure gradient is the lowest; the pressure gradient change near the production well is faster than that near the injection well. To enhance the effect of the gel system and delay its action time, gel should be placed in the area with the lowest pressure gradient between the injection and production wells to plug the formation.
(2). The fluid flow diversion technology of the gel system combination of “injection well + intermediate well” for profile control and flooding increases the oil recovery rate by 7.6% compared to the profile control and flooding of injection wells. The combined fluid flow diversion technology can make the streamline field from the injection well to the deep part of the reservoir spread to both sides of the main streamline until it gradually returns to the main streamline near the wellbore of the production well; it was 16.7% higher than that of the single-injection well profile control and flooding method examined. Due to the increase in injection pressure, the oil washing efficiency in the enhanced swept area increased by 2.3%.
(3). The optimal dosage of the gel system between the injection and production wells is 0.123 PV (equivalent radius of 35 m), which can increase the oil recovery rate by 15.8%. When the dosage of the gel system is small, the fluid can still flow along the main streamline between the injection and production wells, and the effect of expanding the swept volume is not good; conversely, it will lead to excessively high seepage resistance near the gel well, causing difficulties in the flow between the gel well and the production well and affecting the oil washing effect in the swept area in the deep part of the reservoir.
(4). The optimal well placement of the gel well between the injection and production wells is at 1/2 of the distance between them. Both the offset of the well placement toward the production well and the injection well will affect the system’s ability to expand the swept volume and improve the oil washing efficiency. Therefore, it is necessary to select an appropriate well spacing according to the actual situation on the site. When it is impossible to precisely arrange at the center of the injection and production wells, the gel system should be injected near the injection well.

## 4. Materials and Methods

**Figure 8:**
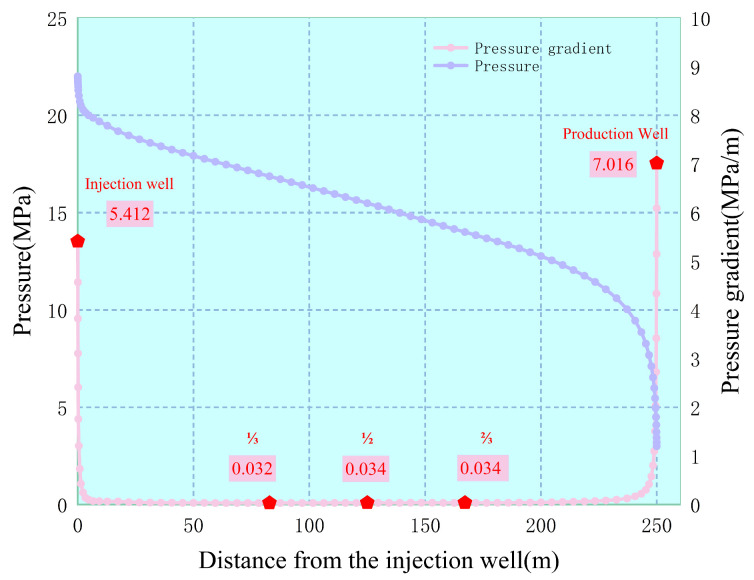
Pressure and pressure gradient distribution curves between injection and production wells.

Based on the deficiencies in the later stage of profile control, the theoretical system of deep fluid flow diversion was further developed with the improvement of profile control technology as the core. As shown in Figure 8, the pressure gradient variation curves near a group of oil and water wells in Block X of a certain oilfield are presented. The physical model was used to calculate the permeability of the reservoir at 800 × 10^−3^ μm^2^, with the injection–production pressure difference set at 18 MPa. The positions of the gel injection wells were respectively set at 1/3, 1/2, 2/3 of the middle section between the injection well and the production well, as well as at the two ends of the injection–production wells. The pressure and pressure gradient at different positions of the gel injection wells between the injection and production wells were subsequently calculated under the condition of water flooding 0.60 PV.

**Figure 9:**
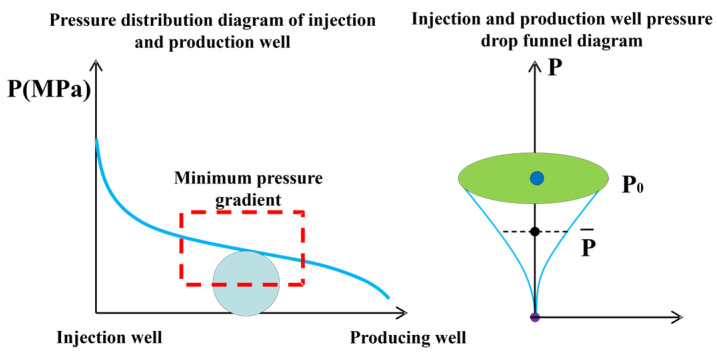
Interwell pressure gradient distribution diagram.

To prevent the injected water from breaking through to the dominant channels, it is essential to determine the appropriate location of the intermediate well. The pressure gradient exhibits a rapid decrease near the injection well and the production well. The pressure drop is mainly concentrated within approximately 10 m around the injection and production wells, suggesting that the pressure changes rapidly at these two locations, with the most energy consumption. Furthermore, the production pressure differential and the distance between the injection and production wells have negligible influence on this conclusion. Therefore, if the blocking agent is to effectively extend the blocking time and prevent it from being broken through by water flow under an excessive pressure gradient, it is necessary to select a suitable position for injecting the blocking agent. As can be seen from Figure 9, the pressure gradient reaches the lowest value at the center of the two wells. If the position of the intermediate well is chosen, this location is the optimal position for injecting the gel system. Here, the gel system is less prone to being broken through and can remain in a stable state for a long time.

The impact of gel well locations on the development efficacy of the “injection well + intermediate well” combined deep fluid flow diversion technology for profile control and enhanced oil recovery was investigated by simulating a typical five-spot well pattern with one injection well and four production wells using a core model. The changes in the oil saturation field of the model were provided by integrating electrode monitoring with the rock–electric relationship curve. The influence of the gel system between injection and production wells on the fluid seepage field was identified based on the variations in the pressure field between the injection and production wells. The mechanism of combined profile control and enhanced oil recovery by reconstructing the seepage field using the gel system between the injection well and the injection–production wells to improve the recovery rate was clarified.

### 4.1. Experimental Setup and Condition

The main apparatuses employed in this experiment were as follows: a confining pressure porosity and permeability meter; an injection system device composed of a high-temperature and high-pressure injection pump, intermediate containers, pipe valves, and fittings; a constant-speed and constant-pressure injection pump device; a simulation system device composed of an intermediate container with water bath pressurization and heating (maximum working pressure of 35 MPa, diameter of 80 cm, 0–95 °C), annular pressure pump, etc.; a water bath pressurization and heating device; a resistance detection system; and other instruments such as vacuum pumps, hand pumps, pressure gauges, electronic balances, stirrers, graduated cylinders, etc.

The experimental chemicals required for this experiment included polymers (HPAM) with a molecular weight of 12 to 16 million (effective content 90%; HPAM is stable below 60 °C but may degrade significantly above 80 °C. After dissolution, HPAM forms a dynamic three-dimensional network structure, where the ‘pores’ consist of the gaps between molecular chains, influencing the solution’s rheology and permeability. When HPAM reacts with crosslinking agents (e.g., Cr^3+^, phenolic resin) to form a gel, a microporous structure is created, typically ranging from nanometers to micrometers (10 nm–10 μm), which can be adjusted by the crosslinker concentration.), Cr^3+^ crosslinker (effective content 90%), stabilizers (effective content 90%), etc. The experimental water was provided by the Third Oil Production Plant of Daqing Oilfield, China; the crude oil extracted from the test area of the Third Oil Production Plant of Daqing Oilfield, China was used. The crude oil underwent dehydration and filtration treatment. The treated crude oil was mixed with aviation kerosene at a mass ratio of 3.5:1 to prepare the simulated oil. The viscosity of the simulated oil was 9.0 mPa · s at a reservoir temperature of 45 °C. The experimental temperature was 45 °C. The injection speed was 2 mL/min. The gel system required 72 h to achieve complete crosslinking. At <60 °C, the gel stability is good, and the life can reach more than 1 year. From 60 to 90 °C, Cr^3+^ crosslinking bonds may be hydrolytically broken and the lifespan shortened to 3 to 12 months. At >90 °C, fast degradation occurs (e.g., 120 °C lifetime may be only a few weeks).

### 4.2. Experimental Steps

(1) Steps for core fabrication

① Ingredient preparation: Prepare the fine quartz sand and cementing substances for the fabrication of artificial cores.

② Sand blending: Mix the quartz sand needed for the model in the prescribed proportion in a square tray and stir evenly. Weigh the chemicals required for preparing the cementing substances separately as per the requirements, blend them, and pour them into the quartz sand for thorough stirring. The stirred sand sample also needs to be sieved to ensure a more homogeneous mixture of sand and cementing substances. Blend the sand in batches according to the formulations for cores with different permeabilities.

③ Mold filling: Before filling the sand into the mold, clean the mold with acetone. During mold filling, it is necessary to ensure uniform sand filling. This is accomplished by moving a sand scraper back and forth horizontally. During this process, the depth of the scraper needs to be constantly adjusted until the sand is uniformly distributed. Then, use a pressure plate to compact the sand.

④ Initial pressure application: Place the mold filled with sand on the pressure testing machine. When placing it, ensure the mold is level and adjust its position to the center line of the pressure plate of the pressure machine. Start the pressure plate vertically, and slowly increase the pressure to the designed value (typically 0.5 MPa). Maintain the pressure for 15 min and then release it. Then, carefully remove the side and end plates of the mold.

⑤ Electrode embedding: Embed the electrodes at the designed positions of the core. One pair of electrodes is embedded at each test point.

⑥ Secondary pressure application: After embedding all the electrodes, place the mold filled with sand on the pressure testing machine. When placing it, ensure the mold is level, and adjust its position to the center line of the pressure plate of the pressure machine. Start the pressure plate vertically, and slowly increase the pressure to the designed value (typically 3 MPa). Maintain the pressure for 15 min and then release it. Then, carefully remove the side and end plates of the mold.

⑦ Heating and solidification: Place the pressed core in an oven for heating and solidification. Set the oven temperature at 85 °C and bake for 6 to 8 h. Then, turn off the oven power and allow for natural cooling and solidification.

**Figure 10:**
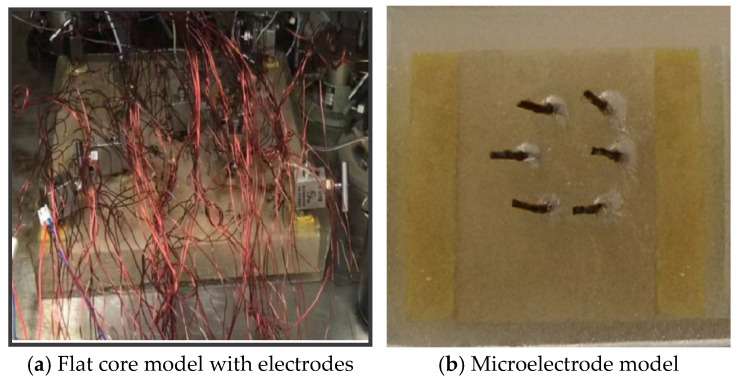
Experimental model diagram.

⑧ Soldering: Use an electric soldering iron and solder to weld the data lines to the electrodes and connect the data lines to the standard resistor to determine the error magnitude and accuracy of the data during the measurement process. The core-making model is shown in Figure 10a.

(2) Establishment methods of rock–electricity relationship of model cores

Using the microelectrode model, a homogeneous model with a size of 4.5 × 4.5 × 4.5 cm^3^ was selected to determine a set of standard curves of resistance and oil saturation applicable to this experiment and to calibrate the resistance and oil saturation. The physical properties of this model are uniform, and its volume is small. The oil saturation at the outlet end can approximately represent the oil saturation of the entire model. The model is shown in Figure 10b.

In the pore medium of the core model, there exist two phases, namely, oil and water. The salt substances in the water phase can ionize into anions and cations. Under the effect of an electric field, the ions will move directionally and generate an electric current, and the current intensity is related to the ion content in the water phase. Assuming that other physical properties in the core model remain unchanged, the resistivity is only a function related to the oil and water content in the pores. Therefore, a standard curve of resistance value and oil saturation can be established. The theoretical method of Archie’s saturation and rock relationship is applied for saturation calibration. Since other components in the core are not electrically conductive, the oil saturation in the core can be directly reflected through different resistance values:

(1) $$I=\frac{R_{t}}{R_{o}}$$

*R_t_*—The resistivity of the rock when it contains oil, Ω·m.

*R_o_*—The resistivity of the rock when it is completely water-bearing, Ω·m.

## Figures and Tables

**Table 1:** Experimental plan for gel well. The location of gel wells and the dosage of gel systems.

Plan	Model Size cm^3^	Effective Permeability of the Model 10^−3^ μm^2^	Model Porosity %	Original Oil Saturation %	Gel Wells		Injection Well	Influencing Factors
					Location of Gel Wells	Gel Injection Volume PV	Gel Injection Volume PV	
1	30 × 30 × 4.5	826	26.0	70.1	/	/	0.15	Dosage of gel system
2	30 × 30 × 4.5	905	26.1	70.8	1/2	0.0628 (25 m)	0.15	
3	30 × 30 × 4.5	816	25.4	70.2	1/2	0.203 (45 m)	0.15	
4	30 × 30 × 4.5	857	26.1	71.1	1/2	0.123 (35 m)	0.15	Location of gel well
5	30 × 30 × 4.5	816	25.2	70.4	1/3	0.123 (35 m)	0.15	
6	30 × 30 × 4.5	884	25.6	70.5	2/3	0.123 (35 m)	0.15	

**Table 2:** Recovery rate and water cut data under different conditions.

Plan	Total Recovery Rate %	Water Cut %	Location of Gel Wells	Gel Injection Volume PV	Influencing Factors
1	46.0	71.1	/	/	Dosage of gel system
2	49.9	70.9	1/2	0.0628 (25 m)	
3	53.7	60.1	1/2	0.203 (45 m)	
4	53.4	63.3	1/2	0.123 (35 m)	Location of gel well
5	54.2	59.9	1/3	0.123 (35 m)	
6	52.5	67.8	2/3	0.123 (35 m)	

## Data Availability

The data are available upon request.
